# Exposure to Subcutaneously Administered Butorphanol in Horses Pre‐Treated With Detomidine or Detomidine‐Vatinoxan

**DOI:** 10.1111/jvp.70051

**Published:** 2026-01-31

**Authors:** Juhana M. Honkavaara, Ninja P. Karikoski, Laura Palvas, Bruno H. Pypendop, Valtteri M. Rinne, Marja R. Raekallio

**Affiliations:** ^1^ Department of Equine and Small Animal Medicine, Faculty of Veterinary Medicine University of Helsinki, Finland Helsinki Finland; ^2^ Department of Surgical and Radiological Sciences, School of Veterinary Medicine University of California, Davis Davis California USA; ^3^ Admescope (Symeres Finland OY) Oulu Finland

**Keywords:** butorphanol, detomidine, horse, subcutaneous, vatinoxan

## Abstract

The aim of the study was to determine the exposure to subcutaneously administered butorphanol in horses pre‐treated with intravenous (IV) detomidine, with or without vatinoxan, a peripherally selective alpha_2_‐adrenoceptor antagonist. Five healthy, adult horses received three IV treatments 7 days apart, in a randomized, cross‐over design: detomidine 20 μg/kg (DET‐B), detomidine 20 μg/kg with vatinoxan 200 μg/kg (DETVAT‐B) and saline (S‐B), all followed by 0.1 mg/kg of butorphanol administered subcutaneously 30 min later. Venous samples were collected between 10 and 270 min after butorphanol administration. Butorphanol concentrations in plasma were analyzed with liquid chromatography coupled with triple quadruple mass spectrometry. Data were analyzed with non‐compartmental methods. The observed areas under plasma butorphanol concentration‐time curves were 8104 ± 2590, 7042 ± 2628 and 5996 ± 1670 ng·min/mL for DET‐B, DETVAT‐B and S‐B, respectively. There were no significant differences between treatments. Pre‐treatment with detomidine, with or without vatinoxan, did not significantly affect the exposure to subcutaneously administered butorphanol in this study.

## 1 Introduction

The impact of sedatives on the exposure to extravascularly administered drugs has not been extensively investigated in veterinary medicine. While it is established that alpha_2_‐adrenoceptor agonists affect the disposition of co‐administered drugs due to their marked hemodynamic effects (Pakkanen et al. 2015; Kallio‐Kujala et al. 2018; Turunen et al. 2020; Paine et al. 2020; Pypendop et al. 2025; Honkavaara et al. 2025), little is known of their impact on the exposure to parenteral, extravascular treatments injected *post hoc*. In clinical settings, drugs can also be administered after the animal is first sedated. In view of this, absorption of subcutaneously deposited treatments could be particularly sensitive to decreases in skin perfusion, caused by drug‐induced vasoconstriction. To that end, tissue perfusion including microcirculation of skeletal muscle, skin and buccal mucosa appears to decrease after alpha_2_‐adrenoceptor agonists in dogs (Pypendop and Verstegen 2000; Niemann et al. 2022; Watt et al. 2025).

Detomidine is the most selective alpha_2_‐adrenoceptor agonist approved for use in horses (Clarke and Taylor 1985). After intravenous bolus administration, an average 20 μg/kg dose of detomidine produces acute cardiovascular changes such as hypertension, bradycardia, atrio‐ventricular blocks, and decreased cardiac output; in healthy horses and ponies, these adverse effects gradually subside during the first hour after detomidine delivery (Sarazan et al. 1989; Wagner et al. 1991; Yamashita et al. 2000). Vatinoxan is a peripherally selective alpha_2_‐adrenoceptor antagonist that does not appear to efficiently penetrate the mammalian blood–brain barrier (Clineschmidt et al. 1988; Honkavaara et al. 2020; Adam et al. 2021). In horses, vatinoxan attenuates the detomidine‐induced increase in vascular resistance and preserves cardiac output while increasing the clearance of the intravenously co‐administered agonist drug (Vainionpää, Raekallio, et al. 2013; Tapio et al. 2018). In accordance, vatinoxan also decreased the exposure to intravenously co‐administered butorphanol in isoflurane‐anesthetized horses premedicated with detomidine (Pakkanen et al. 2015). Conversely, when administered intramuscularly with medetomidine, midazolam, and butorphanol in dogs, vatinoxan increased the early concentrations of the other drugs administered in the same syringe (Kallio‐Kujala et al. 2018).

Butorphanol is a synthetic opioid that is widely used in horses, often in conjunction with sedatives and general anesthetics (Hofmeister et al. 2008; Love et al. 2012). Butorphanol has a wide therapeutic margin and its disposition after subcutaneous administration has been reported in horses (Chiavaccini et al. 2015). It is a relatively small molecule with a large volume of distribution, and is, as such, an attractive candidate in exploring the impact of detomidine pretreatment. The aim of the present study was to investigate the net impact of intravenous pre‐treatment with detomidine, with or without vatinoxan, on the exposure of subcutaneously administered butorphanol in horses. It was hypothesized that detomidine would decrease the early exposure to butorphanol and that adding vatinoxan would prevent that decrease.

## 2 Material and Methods

### 2.1 Animals and Instrumentation

Six, healthy adult horses (three Finnhorse mares, one gelding, and two Warmblood geldings) aged between 6 and 16 years and weighing between 590 and 660 kg were used in this study. The horses were owned by the Natural Resources Institute and Harju Vocational College, and they were housed in groups in a loose housing system. The horses were deemed healthy based on clinical examinations, complete blood cell counts, serum chemistry, and plasma fibrinogen concentration analysis performed prior to each treatment period. In the morning of the study, the horses were fed 1–2 kg of hay with water provided ad libitum. The food was withdrawn 1 h prior to the treatments, and the horses were placed in stocks. The stable was equipped with fan‐assisted ventilation without controlled humidity or temperature. Approximately 30 min prior to the intravenous study treatment administration, a 12 G intravenous catheter (Intraflo, Vygon, Ecouen, France) was aseptically placed into both jugular veins under local anesthesia with approximately 50 mg of subcutaneous lidocaine (Lidor Vet 20 mg/mL, VetViva Richter GmbH, Wels, Austria) per side. The study protocol was approved by the National Animal Experimentation Board of Finland (permit ID ESAVI/7692/2024). The license meets both ARRIVE guidelines and EU legislation criteria. The study was conducted in June 2024.

### 2.2 Study Design and Protocol

This was a three‐period, randomized, cross‐over, assessor‐masked, experimental study. A minimum rest of 7 days between subsequent treatments was implemented to ensure appropriate elimination of the investigated drugs and any relevant carry‐over effect. Randomization was performed a priori into a 6 (horses) × 6 (possible treatment sequences) Latin Square design, with each treatment occurring twice in each period. Treatments were as follows:

SAL‐B: intravenous (IV) saline.

DET‐B: detomidine 20 μg/kg IV (Equisedan 10 mg/mL, Laboratories Syva, Leon, Spain).

DETVAT‐B: detomidine 20 μg/kg IV with vatinoxan 200 μg/kg (Vetcare Lts, Salo, Finland).

Vatinoxan powder was dissolved in sterile, isotonic saline to a 10 mg/mL concentration and each IV treatment adjusted with saline to a final concentration of 15 mL. The treatments were administered as rapid boluses.

All IV treatments were followed 30 min later by 0.1 mg/kg of subcutaneously administered butorphanol (Torpudor 10 mg/mL, VetViva Richter GmbH). Butorphanol was injected (after negative aspiration for blood) into a clipped area superficial to the *m. triceps* at the level of the mid‐humerus using a 20 G, 25 mm hypodermic needle (Kruuse Vet, Langeskov, Denmark) attached to a 5 mL Luer‐Lock syringe (BD Plastipak, Becton Dickinson, Franklin Lakes, NJ, USA). The contralateral jugular catheter was used for blood sampling, while the catheter on the same side had been used to deliver the preceding IV treatments. The injection and sampling sides were alternated between periods. Venous blood samples (6 mL each) were collected into vacuumed tubes containing ethylenediaminetetra‐acetic acid (Vacuette EDTA 6 mL, Greiner Bio‐One, Kremsmunster, Austria). Sampling was always preceded by removal of approximately 3 mL of waste blood from the catheter and followed by a flush with 3–5 mL of heparinized saline. Samples were collected immediately prior to the IV treatment (minus 30 min) and butorphanol (0 min), followed by sampling every 10 min until 150 min. At this point, horses were moved to a stall where they had free access to water and were fed hay based on the degree of any residual sedation. Sample collection continued at 180, 210 and 270 min after butorphanol administration. All samples were put in iced water and centrifuged (2000*g* for 10 min in room temperature). Plasma was then transferred into cryotubes, put in dried ice until stored in a −80°C freezer and later thawed for analysis.

### 2.3 Clinical Observations

Heart rates were counted by auscultation prior to the IV treatment, immediately prior to butorphanol administration and every 30 min until 4 h after the IV treatment. In addition, superficial skin temperatures were simultaneously recorded with a hand‐held infrared thermograph (FLIR E76, Teledyne Flir LLC, Wilsonville, OR, USA) from the site of butorphanol administration as well as from the corresponding, similarly clipped area on the contralateral leg. Ambient and rectal temperatures (Macrolife MT850 Thermometer, Widnau, Switzerland) were recorded at the same time‐points. The investigator evaluating heart rates and temperatures was unaware of the treatment.

### 2.4 Plasma Drug Concentration Analyses

Butorphanol, detomidine, and vatinoxan concentrations were analyzed with a Waters Acquity UPLC liquid chromatograph combined with a Waters XEVO‐TQ‐Absolute triple quadrupole mass spectrometer (Waters, Milford MA USA) in one single batch. Medetomidine was the internal standard. The lower limits of quantification (LLoQ, ng/mL) were 0.4 for butorphanol, 0.1 for detomidine, and 0.4 for vatinoxan. The calibration ranges (ng/mL) were fitted as follows: butorphanol 0.4–4000, detomidine 0.1–2000, and vatinoxan 0.4–2000. Accuracies ranged for all analytes between 85.7% and 101.1% at the LLoQ and between 92.4% and 112.8% above the LLoQ. The precisions were 0.5%–11.4% over the entire range of all calibrations. All concentrations are reported as free base.

### 2.5 Data Analyses

Per a generic power analysis for a paired *t*‐test, six horses were required to detect a 30% difference (standard deviation [SD] of 15%) in the observed area under the plasma butorphanol concentration‐time curve (AUC) between treatments, with a power of 80% and an alpha‐level set at 0.05. Non‐compartmental analyses (with extravascular input for butorphanol) were performed with PKanalix 2024R1 software (Simulations Plus, Lancaster, CA, USA). The highest observed concentration of butorphanol in plasma (*C*_MAX_) and the time of *C*_MAX_ (*T*_MAX_) were observed from the data. For detomidine, only the observed AUC was calculated. The AUCs were calculated with the linear trapezoidal method. All statistical analyses were performed with the SPSS V29.0.2.0 software (IBM Corp, Armonk, NY, USA). The distribution of parametric data was tested for normality using a Shapiro‐Wilks test. Pharmacokinetic parameters were compared between treatments with *t*‐tests for parametric or Wilcoxon Rank Sum tests for non‐parametric data, respectively. For heart rates and temperatures, the effect of time, treatment and their interaction was evaluated with a repeated measures analysis of variance, followed by *t*‐tests for both within and between treatment comparisons. All multiple comparisons were corrected with the Holm‐Bonferroni procedure. Data are presented as mean ± SD for parametric and median (range) for non‐parametric data, respectively. Statistical significance was accepted with alpha < 0.05.

## 3 Results

Data from one horse (horse number 2, “Vili”, in the Supporting Information file B) was discarded due to an apparent treatment failure after DET‐VAT (the vatinoxan concentration was lower by two orders of magnitude compared with all other horses after DET‐VAT). In addition, three out of the five remaining horses had their *C*_MAX_ for butorphanol at the first 10‐min sampling point after S‐B and DET‐B (one horse after DETVAT‐B).

The pharmacokinetic parameters for butorphanol are expressed in Table 1, there were no significant differences between treatments. The AUC for detomidine was significantly lower (*p* = 0.03) after DETVAT‐B (290 ± 93 ng·min/mL) than after DET‐B (422 ± 97 ng.min/mL). Clinical observations and individual plasma drug concentrations for butorphanol, detomidine, and vatinoxan are presented in the Supporting Information data Tables S1 and S2, respectively.

**TABLE 1:** Pharmacokinetic data for subcutaneously administered butorphanol (0.1 mg/kg) from five horses pre‐treated 30 min earlier with intravenous saline (S‐B), detomidine 20 μg/kg (DET‐B) or detomidine 20 μg/kg with 200 μg/kg of vatinoxan (DETVAT‐B).

	S‐B	DET‐B	DETVAT‐B
AUC_0–270_ (ng·min/mL)	5996 ± 1670	8104 ± 2590	7042 ± 2628
*C*_MAX_ (ng/mL)	74.1 ± 39.1	85.1 ± 30.4	78.6 ± 55.2
*T*_MAX_ (min)	10 (10–120)	10 (10–80)	50 (10–80)

*Note:* Data expressed as mean ± SD or median (range). There were no significant differences between treatments.

Abbreviations: AUC_0–270_ observed area under plasma butorphanol concentration‐time curve between 0 and 270 min; *C*_MAX_ = highest observed concentration of butorphanol in plasma; *T*_MAX_ = time of *C*_MAX_.

There were no significant differences in heart rate, ambient temperatures, or superficial skin temperatures between treatments. Rectal temperatures decreased after both DET and DETVAT and remained statistically unchanged after S‐B.

## 4 Discussion

In this study, pre‐treatment with detomidine, with or without vatinoxan, did not significantly affect the exposure to subcutaneously administered butorphanol in horses. The absorption of butorphanol appeared faster than in a previous study, as Chiavaccini et al. (2015) reported a median *T*_MAX_ of 20 min. They injected an equal dose of butorphanol subcutaneously above the supraspinatus muscle, whereas in the present study the drug was administered in the subcutaneous tissue over the triceps muscle at the level of the mid‐humerus. This may at least partially explain the difference between the two studies, since regional blood flow or superficial temperature distribution between the two sites may have differed. Skin temperatures in horses appear to decrease toward the extremities (Soroko et al. 2019). General differences between the two populations of horses or study conditions may also have contributed. Nevertheless, in almost half of the treatments in the present study no upward concentration slope was detected, with the first sample collected 10 min after butorphanol. While this was unexpected, it prevented more detailed assessment of butorphanol's absorption phase. However, a more comprehensive sampling scheme would have been unlikely to significantly affect the overall outcome, as the study would likely have failed in rejecting the null hypothesis of unaffected exposure regardless. While the statistical power was low, the observed means did not suggest that the exposure to butorphanol was reduced in horses pre‐treated with detomidine. On the contrary, the mean observed AUC for butorphanol after DET‐B appeared larger than for the other two treatments.

The results may appear perplexing. The systemic cardiovascular effects of detomidine are well‐described in horses and are comparable with other species of interest (Sarazan et al. 1989; Wagner et al. 1991; Yamashita et al. 2000). While direct evidence from horses is lacking, alpha_2_‐adrenoceptor agonists would not be expected to increase cutaneous blood flow (Watt et al. 2025). Consequently, it would be logical to construe that systemic drug absorption of subcutaneously deposited drugs would be sensitive to changes in skin blood flow and that detomidine would be expected to reduce it. Conversely, vatinoxan would be expected to prevent that effect by blocking the initial detomidine‐induced vasoconstriction (Tapio et al. 2018). That said, casual interpretation of plasma drug concentration data between treatments with opposing effects on clearance may be mistaken, since the amount of drug in the central compartment offers only indirect evidence of its disposition. Detomidine likely reduced the clearance of butorphanol, as reported after intravenous co‐administration in horses (Paine et al. 2020). Thus, a decrease in skin perfusion coupled with a reduction in cardiac output would have created two opposing, but parallel, effects on the exposure to a subcutaneously administered drug, which may explain the present outcome. In a study in rats, vatinoxan decreased the concentrations of medetomidine in plasma measured 15 min after subcutaneous co‐administration, while simultaneously increasing its concentration in the central nervous system (Honkavaara et al. 2025). Nevertheless, accurate quantification of butorphanol's disposition in these horses would have required both an IV butorphanol comparator and measurement of tissue drug concentrations, including sampling of the central nervous system, which was not conceivable in the present study. It is also likely that the peak cardiovascular effects of detomidine had already subsided by the time of butorphanol administration. Moreover, it is presently not known how the systemic cardiovascular effects of alpha_2_‐adrenoceptor agonists translate to changes in cutaneous perfusion in horses. The effect might be less pronounced compared with other species, supported here also by a lack of treatment effect on superficial skin temperature. In dogs, the decrease in skin temperature after medetomidine was obvious, and prevented by simultaneously administered vatinoxan (Vainionpää, Salla, et al. 2013). Vatinoxan decreased the observed AUC of detomidine, which is in accordance with previous reports in horses (Vainionpää, Raekallio, et al. 2013). While the lag time from treatment to sampling prevented more detailed assessment of detomidine's early disposition, vatinoxan is suggested to increase both clearance and volume of distribution of co‐administered alpha_2_‐adrenoceptor agonists via improved hemodynamics.

The heart rates did not differ significantly between treatments in the present study. This is in clear conflict with the known, severe bradycardic impact of detomidine in horses. While no other cardiovascular variables were monitored, detomidine‐induced bradycardia was expected to be pronounced and differ from the other two treatments, even with the very small sample size. It is difficult to explain the lack of any treatment effect, particularly since both detomidine and vatinoxan concentrations in plasma from the present study were comparable with previous studies, where marked effects of treatment on heart rate were also reported, despite conservative *post hoc* corrections (Vainionpää, Raekallio, et al. 2013; Tapio et al. 2018). In the present study, heart rates were obtained by auscultation of the ventral thorax for 30 s, a commonly used and acceptable method. However, peripheral pulse rates would have been a practical method to confirm dynamic cardiac frequency. It is also worth noting that heart rates were not recorded for the first 30 min after the IV treatments and some of the initial effects may have already lessened (Wagner et al. 1991; Vainionpää, Raekallio, et al. 2013). Assessing oscillometric arterial blood pressure, oral mucosal color and capillary refill times would have offered additional information on the horses' cardiovascular status during the observational period.

The results of this study should be considered in view of several limitations. First, due to deficient early sampling, the absorption phase for butorphanol was not identified. Secondly, the hypothesis was practically irrelevant for horses. In fact, the research question was formulated based on clinical events in small animal practice, where subcutaneous injections of drugs can be administered to dogs and cats that have been sedated with alpha_2_‐adrenoceptor agonists, such as medetomidine or dexmedetomidine. Extrapolating the study question into equids was justified, as a comprehensive investigation of a relatively unknown *T*_MAX_ requires frequent sampling over an extended period of time, which could have resulted in unacceptable blood loss in smaller animals. However, the anatomy of the subcutaneous space may not be comparable between species, so the outcome of a similar study in dogs or cats may well differ from the present results. Furthermore, a clinically more relevant drug, such as a non‐steroidal anti‐inflammatory drug, was not investigated due to a concern for potential tissue inflammation in horses, especially after repeated subcutaneous administration. Instead, butorphanol was chosen since its subcutaneous administration had been investigated in horses and is generally well tolerated. The statistical power was also reduced by loss of one complete data set, which may have impaired detecting significant treatment effects. Finally, the outcome of this study only applies to the studied doses of drugs in a similar population of horses.

In conclusion, the exposure to subcutaneously administered butorphanol was not significantly affected by pre‐treatment with detomidine or detomidine‐vatinoxan in horses. Frequent early sampling is advocated for drug absorption studies.

## Author Contributions

**Juhana M. Honkavaara:** study design, data collection, formal analysis, original draft; **Ninja P. Karikoski:** study design, data collection, reviewing and editing; **Laura Palvas:** study design, data collection, reviewing and editing; **Marja R. Raekallio:** study design, data collection, reviewing and editing; **Valtteri M. Rinne:** drug concentration analyses, reviewing and editing; **Bruno H. Pypendop:** formal analysis, reviewing and editing. All authors read and approved the submitted versions.

## Ethics Statement

The authors confirm that the ethical policies of the journal, as noted on the journal's author guidelines page, have been adhered to and the appropriate ethical review committee approval has been received. The authors confirm that they have adhered to European standards for the protection of animals used for scientific purposes. The study was approved by the National Animal Experimentation Board of Finland (permit number ESAVI‐33905‐2024).

## Conflicts of Interest

J.M.H. has received funding in the past from Vetcare Ltd. J.M.H. and M.R.R. are named among the inventors in a patent concerning vatinoxan; patent holder is Vetcare Ltd. All other authors declare no conflicts of interest.

## Supporting information


**Table S1:** Clinical observations from five horses treated at baseline with intravenous saline (S‐B), detomidine 20 μg/kg (DET‐B) or detomidine 20 μg/kg with 200 μg/kg of vatinoxan (DETVAT‐B), followed by subcutaneously administered butorphanol 30 min later.

**Table S2:** Plasma drug concentrations from five horses treated with 20 μg/kg of intravenous detomidine (DET‐B), 20 μg/kg of detomidine with 200 μg/kg of vatinoxan (DETVAT‐B) or saline (S‐B), followed 30 min later by 0.1 mg/kg of subcutaneously administered butorphanol.

## Data Availability

The data that supports the findings of this study are available in the Supporting Information of this article.
