# Supplementary material for: Exposure to Subcutaneously Administered Butorphanol in Horses Pre‐Treated With Detomidine or Detomidine‐Vatinoxan
Source: J Vet Pharmacol Ther. 2026 Jan 31;49(3):289–94. doi: 10.1111/jvp.70051 (PMC13159762; doi:10.1111/jvp.70051)
Supplement: Supplementary file 1 — Table S1: Clinical observations from five horses treated at baseline with intravenous saline (S‐B), detomidine 20 μg/kg (DET‐B) or detomidine 20 μg/kg with 200 μg/kg of vatinoxan (DETVAT‐B), followed by subcutaneously administered butorphanol 30 min later. [file JVP-49-289-s001.docx]

Table S1. Clinical observations from five horses treated at baseline with intravenous saline (S-B), detomidine 20 μg/kg (DET-B) or detomidine 20 μg/kg with 200 μg/kg of vatinoxan (DETVAT-B), followed by subcutaneously administered butorphanol 30 minutes later.

|  | Treatment | Baseline | 30 min | 60 min | 90 min | 120 min | 240 min |
| --- | --- | --- | --- | --- | --- | --- | --- |
| Heart rate  (1/min) | S-B | 50 ± 11 | 39 ± 4 | 42 ± 13 | 40 ± 13 | 44 ± 15 | 46 ± 12 |
|  | DET-B | 45 ± 8 | 33 ± 5 | 35 ± 7 | 33 ± 8 | 37 ± 8 | 40 ± 7 |
|  | DETVAT-B | 53 ± 8 | 38 ± 9 | 36 ± 10 | 40 ± 13 | 41 ± 9 | 43 ± 12 |
| Rectal temperature  (ºC) | S-B | 37.7 ± 0.2 | 37.8 ± 0.2 | 37.9 ± 0.3 | 37.9 ± 0.2 | 37.9 ± 0.2 | 37.8 ± 0.3 |
|  | DET-B | 37.7 ± 0.3 | 37.8 ± 0.3 | 37.6 ± 0.5 | 37.2 ± 0.6 | 37.1 ± 0.4 | 37.5 ± 0.4 |
|  | DETVAT-B | 37.7 ± 0.3 | 37.7 ± 0.2 | 37.2 ± 0.2 | 36.9 ± 0.5 | 36.9 ± 0.5 | 37.5 ± 0.5 |
| Injection site skin temperature  (ºC) | S-B | 28.1 ± 1.8 | 28.1 ± 3.6 | 28.3 ± 3.2 | 27.8 ± 3.4 | 28.4 ± 3.0 | 29.3 ± 2.1 |
|  | DET-B | 28.1 ± 3.3 | 26.7 ± 2.6 | 27.2 ± 1.9 | 26.6 ± 1.9 | 26.2 ± 1.9 | 27.7 ± 2.8 |
|  | DETVAT-B | 27.5 ± 2.7 | 26.8 ±2.6 | 28.5 ± 1.1 | 26.8± 2.1 | 27.3 ± 1.2 | 29.4 ± 1.4 |
| Contralateral skin temperature  (ºC) | S-B | 27.8 ± 2.1 | 27.7 ± 2.1 | 26.7 ± 2.3 | 27.2 ± 2.4 | 27.0 ± 2.1 | 29.2 ± 1.2 |
|  | DET-B | 27.5 ± 3.5 | 25.6 ± 2.3 | 26.2 ± 1.9 | 25.0 ± 1.3 | 24.7 ± 0.7 | 27.3 ± 2.8 |
|  | DETVAT-B | 27.5 ± 2.5 | 27.5 ± 2.5 | 26.9 ± 0.8 | 24.8 ± 1.4 | 24.9 ± 1.6 | 27.4 ± 2.7 |
| Ambient temperature  (ºC) | S-B | 18.0 ± 2.7 | 19.2 ± 2.1 | 18.8 ± 2.4 | 19.4 ± 2.2 | 18.6 ± 2.6 | 20.2 ± 2.6 |
|  | DET-B | 18.0 ± 2.8 | 18.2 ± 3.0 | 18.5 ± 2.6 | 18.8 ± 2.7 | 18.9 ± 2.6 | 19.7 ± 2.9 |
|  | DETVAT-B | 18.0 ± 2.9 | 18.5 ± 2.9 | 18.7 ± 2.7 | 18.9 ± 2.8 | 19.1 ± 2.8 | 20.3 ± 2.8 |

Significantly different from baseline (p < 0.05) when underlined. Time-points 150 and 180 min were excluded from the table (there were no significant changes in the presented variables over that time period).
